# Infra Low Frequency Neurofeedback Training for Trauma Recovery: A Case Report

**DOI:** 10.3389/fnhum.2022.905823

**Published:** 2022-08-01

**Authors:** Hanno W. Kirk, Monica Geers Dahl

**Affiliations:** ^1^National Association of Social Workers, Washington, DC, United States; ^2^Geers Hypnosis LLC, Inverness, FL, United States

**Keywords:** trauma, PTSD, Neurofeedback, biofeedback, relapse prevention, torture and Neurofeedback, mTBI and Neurofeedback, alcoholism and Alpha Theta training

## Abstract

This paper reviews how and why ILF Neurofeedback has proven to be a parsimonious and efficient way to remediate the neuro-physiological effects of trauma. Reference is made to several large- and small-scale institutional proof of concept experimental studies each addressing a specific kind of trauma. It ends with a case report by the author (Kirk) working with an American combat veteran. It makes the argument that given its success that ILF Neurofeedback and Alpha-Theta training become accepted as part of an integrative and holistic approach for treating survivors of trauma.

## Introduction

Trauma affects the brain at all levels. Basic homeodynamic rhythms and the self-regulatory capacity of the brain are impaired (Fisher, 2014). Equally important is that trauma “memories” become encoded diffusely in our physiology (van der Kolk, 2014). The degree of traumatically induced change is determined by the severity, complexity, repetition, duration of the trauma experience(s), and most importantly, by the vulnerability of the particular nervous system. Hence traumatic events cannot be appraised in isolation. They must be seen in the context of the victims' entire history, which collectively determines their vulnerability.

Sources of trauma can vary widely. Developmental trauma in childhood can be from attachment issues, neglect and abandonment, lack of safe environment, or physical, emotional, sexual abuse. Developmental trauma may leave a life-long imprint of not having an integrated sense of self (Gerge, 2020), a poor sense of the other, often not living in their body, or having a poor body image, and living in a constant state of fear (Fisher, 2014). For teens or adults, exposure to violence, whether physical, emotional, or mental will likely leave an imprint of permanent sense of loss of trust and safety, and a constant state of fear and vigilance. Natural disasters, accidents, illness, torture, sexual assault, death of a loved one, even loss of a relationship impact all ages, possibly triggering a traumatic response. The hypervigilance engendered by the shift to fear/anxiety based limbic processing leads to a state of constant over-arousal in the right hemisphere and limbic system (Amen, 2004).

These changes interfere with a person's cognitive processing of the traumatic events (Lee et al., 2021). As Bessel van der Kolk observed, “All trauma is preverbal” (2014, p 243). When the sympathetic nervous system is hyper-activated, the ability to speak and describe the experience can be impaired (Kano and Fukudo, 2013; Dahl, 2020).

In its more pronounced clinical manifestation, the inability to verbalize the experience is given the label of alexithymia. This is likely to also involve an inability to identify emotions in the present (Frewen et al., 2008).

Trauma histories tend to be fragmented. They are not remembered by the brain as a story with a beginning, middle, and end. Instead, trauma is experienced in a non-sequential, emotionally loaded fashion. Isolated sensory imprints, fragmented images, sounds, smells, and physical sensations are usually accompanied by intense emotions that can range from terror and rage to helplessness. Trauma histories tend to come out in bits of disjointed sensory experience because the declarative, explicit memory is not accessible.

When the original trauma experience(s) involved overwhelming pain, fear, or terror, the brain may suppress the recall of the memory. This means that the mind can block out bodily sensations, and a sense of leaving the body can occur for the duration of the experience. A person struggling with these impairments may be diagnosed with a condition of depersonalization or dissociation, Dissociative Identity Disorder, (Lanius et al., 2010), Borderline Personality Disorder, or even psychosis (Schore, 2003; Fonagy and Luyten, 2015). Some people are so dissociated that they cannot relate to their body except as an object fit for punishment, often by self-harming actions. Some people go on to develop a lifelong habit of dissociation to situations that trigger a fear-based limbic response (Busuttil, 2004). These people appear to be stuck in a past dominated by fear. When a person has lost a sense of self and is unable to have a good sense of others, this entails disruption of the natural process of maturation of the default mode network (DMN) (Bluhm et al., 2009; Sripada et al., 2012). The consequence is a distorted narrative about the self (Fisher, p 214).

The brains of many traumatized people are trapped in rigid response patterns (Gerge, 2020). The most characteristic and troublesome symptom of PTSD is that of re-experiencing the trauma. Anything that triggers the flashback evokes the original whole-system response to the trauma in all its particulars. By virtue of the salience of the original trauma, the entire event is registered in the body-mind as a unitary memory. Subsequent recall of the event then involves the whole memory; the specific, explicit “event memory” and the accompanying implicit “state memory.” This richly embedded set of memorized responses is diffusely registered throughout the body (van der Kolk, 2014). It is grounded in our most basic survival mechanisms. There is value in the trauma being remembered permanently, so in that sense, the system is working as designed.

When there are successive multiple trauma experiences, the body-mind eventually accommodates to a perpetual state of anticipating threat. This places a great burden on the person, with high costs to the person's well being and everyday functioning. This state of chronic threat awareness and readiness to respond (fight/flight/freeze) typically defies standard therapeutic attempts to achieve its extinction (Othmer and Othmer, 2009; Lake, 2015). These are whole-body memories centered in the limbic structures and the autonomic nervous system, making them inaccessible for processing and resolution via the analytical mind for rational examination.

## Neurofeedback as Therapeutic Tool for PTSD

Cognitively-based therapies are of little or no utility the dysregulated brain has been restored to functional self-regulation, and the visceral hyper-reactivity has been assuaged or extinguished. It is proposed that the first task of trauma therapy should attend to calming fear-driven over-arousal (Fisher p 70). Neurofeedback has proven to be a parsimonious and efficient system to address this primary task.

The assumption underlying Neurofeedback is that traumas are encoded in functional brain networks not only as memories but in their physiological aspects (Othmer and Othmer, 2020). *Via* the autonomic nervous system, the trauma memory is registered in peripheral physiology as well. The peripheral indicators of unresolved trauma can be observed in autonomic response patterns, as well as in hand, feet, neck, head activity and facial tics. The regulation of physiological arousal is intimately associated with the regulation of the autonomic nervous system (ANS). Treatment for PTSD involves restoring a proper balance between the sympathetic and parasympathetic branches of the ANS. Both arousal regulation and autonomic regulation are in turn intimately associated with the regulation of the affective domain.

This phenomenon can be better understood in terms of functional alteration rather than fundamental structural damage to the system. It has also been learned that memory is not absolute, but contingent, and it can be retroactively modified. Ideally, the work of trauma recovery is one of transitioning the trauma experience from one that is viscerally felt in all bodily systems, to one that appropriately resides in historical memory, like all other recallable events in one's life (Othmer and Othmer, 2021).

Taking advantage of neuroplasticity (Schwartz and Begley, 2003), Neurofeedback aims at the systemic restoration of the capacity to live out of a calm, relaxed, and well-controlled state. This is best accomplished under benign conditions, without any reference to the trauma history. Infra low frequency (ILF) Neurofeedback is ideally suited to this task. Through the individualized training, conducted at the optimal response frequency (ORF), Neurofeedback provides a ready means of training arousal regulation in a controlled fashion for improved functionality.

In ILF training, the guiding principle is the brain's hierarchy of regulation. As in any self-regulating system, stability of brain function is the priority, but particularly so by virtue of the above considerations. The second priority is the quality of arousal regulation, to which the right hemisphere gives us preferential access. By targeting the right parietal region, we can achieve physical calming. With trauma clients, the training for self-regulation is begun with first placement at T4-P4.

The right hemisphere is also dominant for the organization of our affective domain. Emotional calming is achieved with right pre-frontal training, at T4-Fp2. Much of PTSD symptom presentation involves emotional reactions, predominantly anger and rage, but also sudden onset of feelings of fear, or terror. Schore (2003) showed that the right prefrontal or orbital cortex areas are the most affected when fear-based processing becomes predominant. This brain region is of critical importance in the top-down regulation of affective states (Ghashghaei et al., 2007). To restore emotional impulse control, and to calm and stabilize a person struggling with anger/rage outbursts, anxiety, or fear reactions, a right prefrontal placement at T4-Fp2 is therefore used, in addition to right parietal placement at T4-P4 (Othmer, 2019).

Along with calming the brain with right hemisphere training, we need to promote overall system stability. This has been found to depend on the coordination of the left and the right hemispheres. For this purpose, interhemispheric placements at homologous sites are used in bipolar montage. In practice, the protocol largely defaults to training at T3-T4.

In their effort to control their sense of loss of personal agency during their trauma experiences, some persons with PTSD develop obsessive-compulsive traits. For this a left prefrontal placement at T3-Fp1 is used. It is common to end sessions on the T4-P4 montage to reinforce the sense of place in the world and of comfort in the body, as well as to augment right-hemisphere calming (Othmer, 2019).

For persons who are either feeling self-alienated or not aware of the Self, a third option, can be added to the Neurofeedback process: ILF Synchrony training. It is suggested to be used after the ILF bipolar training, described above, has calmed down the affective over arousal, and interhemispheric stabilization has occurred. The electrode placement for this is at FZ + PZ at a frequency of nominally 0.05 mHz. The motivation was to impinge on the coordination of the frontal and parietal hubs of the DMN. This relatively new two-channel sum training has been found to produce calm self-awareness (Othmer, 2019), and a return to the capacity for happiness^1^.

A fourth option for using Neurofeedback with trauma patients is Alpha-Theta (A-T) training. This protocol provides for reinforcement on alpha and theta band activity—whichever is dominant in the moment. The intent is to induce calm yet wakeful states that facilitate the non-verbal processing of psychological aspects of the traumas in an atmosphere of safety, and in the absence of intrusions from the trainee's own self-critical faculties. For persons with PTSD, A-T training is an essential component, but it needs to be undertaken with great care. The general rule is that it be done only after hypervigilance and over arousal has been calmed and interhemispheric stability has been obtained. Persons who are still hypervigilant and unable to relax will not benefit from standard A-T training. Without sufficient calming, A-T training might trigger instabilities or even abreactions (Wiedemann, 2020). Indeed, as William Scott found during the Cri-Help study (see below), a subset of trainees became more agitated with A-T training. What these persons had in common was high amplitude (>12 μV) in their Alpha band activity. Using one-channel down-training at PZ-A1 he was able to lower their arousal level (Scott, 2017). In general, the rule is that when a person does not benefit from A-T training, a return to calming and stabilizing with ILF is called for.

## Experimental Programs in Neurofeedback for Treating Trauma

No discussion of Alpha-Theta training is complete without reference to Eugene Peniston. Starting in 1979, Peniston applied a multimodal protocol developed at the Menninger Foundation to the treatment of alcoholism among Vietnam veterans at the Ft Lyon, Colorado, Veteran's center (VA). The components of his protocol included pre-training in temperature biofeedback and up to 30 Alpha-Theta training sessions with “scripted” guided imagery. Suggestions for a relaxation induction were followed with specific suggestions for desired behavioral changes such as alcohol-rejection and anger management (Peniston and Kulkosky, 1989). The hypothesis was that biofeedback in the form of hand warming would lead to a systematic desensitization. An 18-month follow-up showed that all the participants in the experimental-group were still abstinent. Indeed, they were all still abstinent 8 years later, whereas all participants in the control group had relapsed into alcohol use. Along with this astounding success rate for abstinence Peniston found that there was a surprising reduction in PTSD symptoms (Peniston and Kulkosky, 1991).

Peniston then started to focus on using the same multimodal approach to aid veterans in decreasing PTSD symptoms as well as treating them for alcoholism. After several more carefully structured experiments, using what became known as the Peniston-Kulkosky protocol, the results were documented with several outcome measures. At the conclusion of their 1991 experiment (N29) significant changes toward normalization in the Minnesota Multiphasic Personality Inventory (MMPI) were seen in the experimental group, along with a decrease in psychotropic medication and a decrease in anxiety-provoking dreams or nightmares. The experimental group showed a marked reduction in the following subscales: depression, hypochondria, hysteria, psychopathic deviation, paranoia, psychasthenia, hypomania, introversion, and the PTSD subscale.

Peniston's ground-breaking studies kindled several other pioneering experimental programs using Neurofeedback for treating various kinds of trauma. The group at EEG Spectrum clinic, then in Encino, California, developed an altered protocol which relied on SMR-beta Neurofeedback as preparation for the Alpha-Theta sessions (Scott, 2017). The bias in this choice was toward taming impulsivity and enhancing pre-frontal inhibitory control. The assumption was that a well-regulated brain would be able to exercise good judgment, “thus paving the way for mastery over addiction” (Othmer and Othmer, 2021). The protocol consisted of Beta1 training at C3-Fpz (i.e., 15–18 Hz), in combination with SMR training at C4-Pz (12–15 Hz). Lost in the bargain was Peniston's thermal biofeedback, which targeted autonomic nervous system regulation. However, introducing beta and SMR training restored access to the autonomic nervous system (Scott et al., 2005).

The new protocol was used in a proof-of-concept study at CRI-Help, a large residential treatment center in North Hollywood, California. There were 121 participants, divided into experimental and control groups with matched addiction severity index.

Success in training was documented *via* improved retention in program, normalization of Continuous Performance Test scores (TOVA), and improvements on the MMPI. Relapse prevention was assessed at 1 year, favoring the experimental group by a factor of 3:1 (referenced to entry in program, where the groups were matched in terms of addiction severity index). At 3-year follow up, the control group had continued to attrition into relapse whereas the experimental participants had maintained their sobriety. Again, the surprise finding, mirroring Peniston's findings, was that the training had facilitated the resolution of comorbid trauma syndromes found to have a high incidence in this population.

Some eight U.S. military bases adopted the use of Neurofeedback protocols for active-duty service members in the 2009 time frame and after, and the training was also offered in Afghanistan and Iraq (Lake, 2015). At Camp Pendleton, CA, symptom tracking for 65 categories of interest was conducted on the first 300 active-duty combat Marines to experience the training in the 2009–2010-time frame. This was the first large-scale insertion of the then-novel Infra-Low Frequency protocols into an institutional setting. The data set revealed resolution to beneath clinical significance in 75–80% of trainees for most categories tracked. Best response was observed for migraine reduction [92% responders], depression [81%], anxiety [77%], whereas tinnitus had the lowest response rate [49%]. Suicidality became a non-issue with the few who had listed this concern (Villanueva et al., 2011). The outcomes reported include overall improved regulation and enhancement of cognitive functions. A collateral benefit of Neurofeedback was that the often comorbid minor traumatic brain injury (mTBI) was addressed simultaneously even as PTSD was the primary concern.

Following up on the theory that ILF NF could be used for mTBI, (Carlson and Ross, 2021) conducted a pilot study at the VA Center in Hawaii on veterans suffering with post concussive symptoms from IED blasts during their combat tours. Based on the success of the pilot study a follow-on clinical study with a larger number of participants has been funded (Carlson personal communication 3/15/22). Target date for completion is 2023.

The Red Cross in Sweden conducted an experiment of 10 ILF NF sessions with “traumatized” refugees who had been exposed to war-related torture and had failed to respond to conventional therapies. The researchers concluded that ILF Neurofeedback was a viable tool in restoring a sense of identity, helpful for rebuilding a life after war related torture and refugee status (Nilsson and Nilsson, 2014). A subsequent study extended the training from 10 to 20 sessions midway through the study, yielding major progress of symptom abatement during the second group of 10 sessions. Overall average reduction in mean symptom severity was >50%, and no plateauing of progress was in evidence at 20 sessions (Metso and Duberg, 2016). A small study in Australia of refugee torture victims yielded similar positive results (Askovic et al., 2017).

Each of the above briefly described experimental proof of concept studies have been reported in the literature. They collectively support Neurofeedback as a highly effective tool for remediating the profound systemic changes caused by a wide range of trauma experiences. Largely unreported are the experiences of private practitioners of ILF Neurofeedback who have been working with trauma victims for many years. Over the last 16 years, this author (Hanno Kirk) has used ILF Neurofeedback coupled with Alpha-Theta training with clients who had experienced a wide range of traumas. These included severe sexual or physical abuse as a child, domestic violence abuse, survivors of natural disasters or car crashes in which family members died, and veterans who witnessed carnage in war zones. The author is a member of a network of NF therapists who committed themselves to providing Neurofeedback training to veterans with PTSD at no cost^2^.

## A Case Report

What follows is a report by the first author of a 55 year old US Army veteran who had been diagnosed by the VA with combat related PTSD. His background included having graduated from the US Military Academy at West Point, the American army's premier training school for officers. After 10 years of active duty in various non-combat assignments, he left active service for law school. While building a career in law over the next 20 years, he remained active in the US Army Reserve and National Guard. In 1990 during Operation Desert Storm his National Guard Military Police Unit was deployed to Saudi Arabia to handle Iraqi prisoners of war. His unit was again called up for active duty during the Balkan wars in 1995, this time for base security of NATO installations. Since neither of these two active-duty assignments had any combat components, he returned each time physically and mentally in good shape.

Due to his unique background in the law and training skills, he was recalled with rank of Colonel to active duty in 2008. He was tasked to command a Task Force to train Afghan Police units to be law enforcement officers in rural Kandahar Province. This was the most contested part of Afghanistan and the twelve 15-man training teams, and their trainees often came under fire. In contrast to combat units in the area, his teams had not had casualties for the first 11 months of their 1-year tour of duty. Indeed, the men in his task force came to believe that they were in a divine protective bubble. Then just 2 weeks away from the end of their tour in Afghanistan “the shit hit the fan” (his words), and two of his men lost their lives while five more were seriously wounded. He felt responsible for their deaths and berated himself for exposing them to danger “needlessly” before their scheduled return home. In what is known as survivor guilt (Kubany et al., 1996), he felt he should have been killed instead of the men. He felt especially guilty about the death of one his most valuable non-commissioned officers, who drowned when his Humvee ran off the road during a night mission and overturned in an irrigation canal.

He said that initially he had tried to “sort things out” by himself for a year, thinking that with time he would get better. When his symptoms persisted, and he feared that his wife would leave him, he signed up for standard care with the two nearest Veterans Administration (VA) centers. He was promptly diagnosed with PTSD and comorbid depression and anxiety. He went to weekly individual counseling, and twice weekly support group meetings with fellow veterans with PTSD. He was also prescribed a handful of medications for depression, anxiety, ADHD, and sleep.

After 2 years, and seeing little improvement in his PTSD symptoms, he embarked on an online search for alternatives. He came upon Neurofeedback as a remedy for PTSD and found that I was the only VA-accredited therapist providing this intervention in West Virginia. Because the VA was not offering this therapy in-house, he applied for a referral to me through the Community-Based Care program. After getting VA approval we began Neurofeedback training on May 25, 2012.

On intake, he filled out the PCL-5, the standard instrument used by the US military to assess PTSD. His score was 71, which falls into the category of moderate to serious. To further assess his PTSD as well as other issues, he also filled out the 150 item Symptom Rating Scale^3^. The high-rated symptoms relating to PTSD were “nightmares, flashbacks, anxiety attacks, sleeplessness, migraines, angry outbursts, problems with focusing at work, depression, and lack of connectedness.” In addition, he listed “numbing, problems of relations with coworkers and his wife, and pretty much everything.”

Evidence of his determination to recover was that he committed himself to 10 Neurofeedback sessions in 5 weeks. Although working full time as Assistant Prosecuting Attorney, he faithfully made the 3-h roundtrip twice weekly and attended all 10 sessions. Each session was about 50 min long and consisted of debriefing on inter-session progress by reviewing the identified major symptoms. This was followed by 30 min of ILF Neurofeedback. The primary placements were at T4-P4 for calming his hypervigilance and high arousal issues, followed by T3-T4 for system stability. To restore prefrontal control of his emotions, especially anger and rage outbursts, we used T4-Fp2, followed by T4-P4. He made rapid progress, as attested to by his own inter-session reports, and his wife. She came in at the fifth session, wanting to know what we were doing because, “I now have my husband back.”

After the tenth session on June 29, he retook PCL-5 and his score was 59; a reduction of 12 points in 5 weeks^4^. He claimed that his most troublesome symptoms had disappeared and that he was titrating off his medications. He penned a testimonial saying that he had received more benefit from 10 sessions of Neurofeedback than from 2 years of VA care. He e-mailed it to each of his VA therapists and prescribing doctors. However, he knew he was not “cured,” and so he opted to continue with the Neurofeedback sessions, though at lesser frequency. First, we reduced it to twice a month, then to once a month as his work duties as Assistant Prosecuting Attorney increased. Being busy kept him from ruminating on his survivor guilt and he continued to see progress in his day-to-day activities and behaviors. He was still having some nocturnal flashbacks and nightmares. That was a sign that we needed to work on resolving the deep-seated trauma memories.

We added two channel sum training at Fz + Pz at 0.05 mHz, and this was helpful for him. His wife reported that a drunken driver had crashed his car into the stone retaining wall in front of their house, but that her husband wouldn't bother to get up, saying the police could handle it. She commented that if this had happened before he started Neurofeedback, he would have been up immediately with the two guns he kept under his pillow to “deal with the threat.” Since nightmares were still occurring, albeit infrequently, this indicated that the deep-seated state memories of the trauma had not been resolved. He also reported that he was still uncomfortable in crowds and avoided social gatherings. “Suspicious looking packages” still triggered a physiological arousal response.

**Figure 1:**
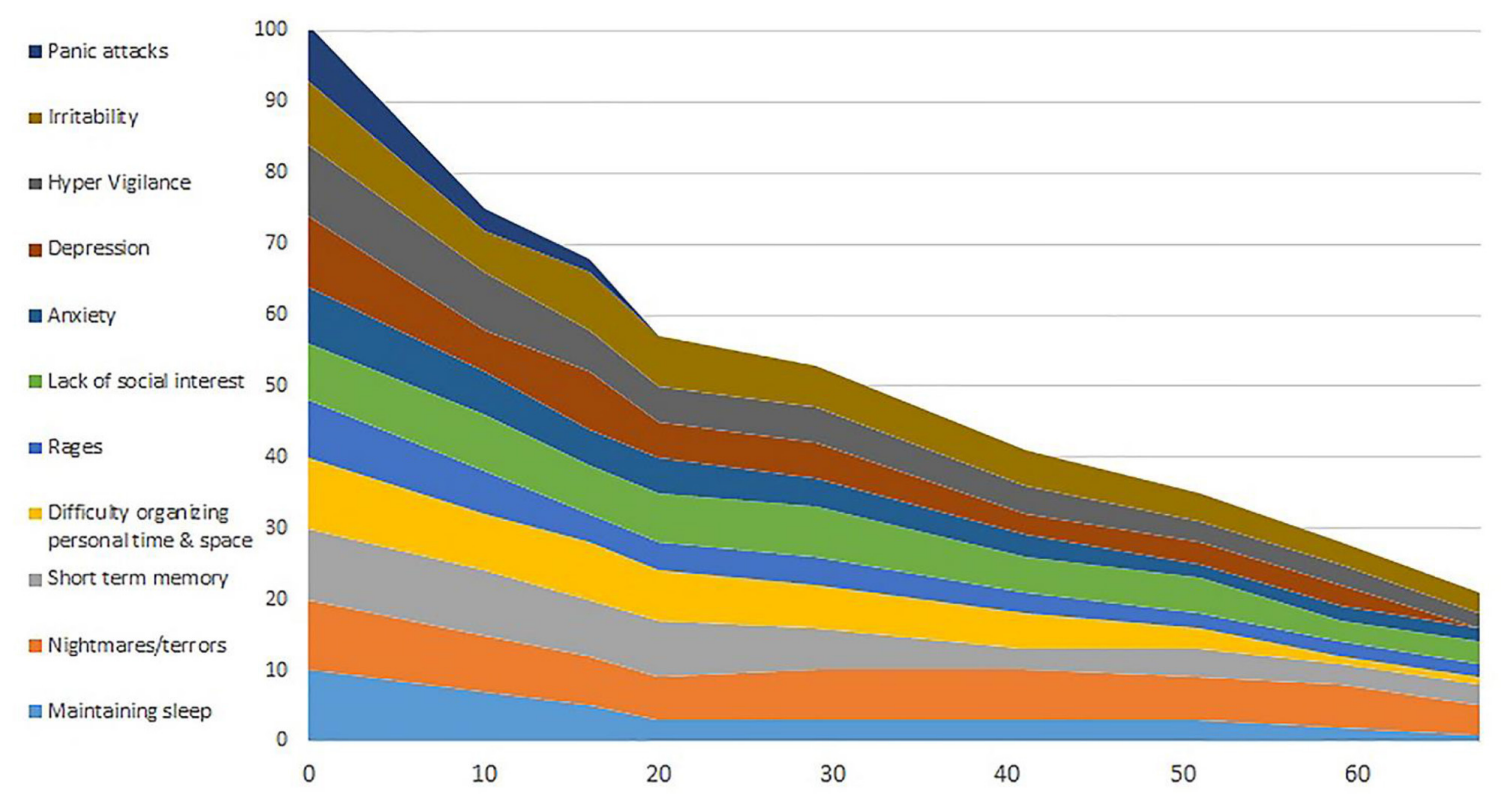
Results of symptom tracking over sessions, which extended over several years. The EEG expert symptom-tracking program was used (www.eegexpert.net).

We decided to add two-channel sum Alpha Theta training with alpha frequency set at 10 Hz and theta set at 7 Hz, to aid in psychological resolution. There is a choice of scalp locations for this kind of training. In his case, after successful calming and restoring brain stability, we used P3 + P4, alternating from session to session with O1 + O2. A recorded guided imagery induction was added to engender a deep relaxation state. After one session, he said that he had “seen” the trauma sequence during his session, which he described as “movie scene without the emotional soundtrack.” Immediately after the session he was able to calmly verbalize what had happened during the trauma incident for the first time. This was evidence that he had successfully decoupled the state memory and could now see the historical memory without triggering physiological arousal (See Figure 1).

This veteran continued a schedule of monthly “refresher” sessions. Eighteen months into his rehabilitation from PTSD, he announced that was going to campaign for the position of Prosecuting Attorney, which he won. He acknowledged to me that he could never have campaigned or spoken in front of crowds prior to Neurofeedback training. He and his wife have continued to come for occasional joint sessions to “keep the stress down.”

In summary, this client was highly motivated to overcome the burden of his PTSD symptoms. This is not true for all the veterans I have trained with Neurofeedback. Some who live at some distance were unwilling to commit themselves to the intensive regimen to which this veteran agreed. Other veterans who depend on the VA disability checks had quit therapy because they feared losing their benefits if they got well. The determination and resiliency of this veteran, his trust in me, and faith in the method clearly contributed to his success. As the training proceeded, I could observe the visible change from the man who appeared pained, depressed, and hopeless when he first came in, to the person who found life to be interesting, challenging, and enjoyable. He and his wife have revitalized their marriage. They display affection and an easy back and forth humorous banter every time they come in for a “refresher session” more than 10 years after his initial visit.

## Neurofeedback: the Case For Evidence-Based Status

Neurofeedback is a non-invasive behavioral training that trains neuronal networks to improve self-regulation. It thereby ameliorates symptoms of PTSD while simultaneously reducing comorbidities. The above brief review of select programs are proof of concept that addictions, PTSD from combat or from torture, as well as mTBI and their dysregulating sequelae can be successfully remediated with the combination of ILF Neurofeedback and Alpha-Theta training. Each of the programs reviewed had shown positive results in a relatively short time along several dimensions:

- Neurofeedback dramatically improves retention in existing drug/alcoholism rehabilitation programs, reduces recidivism, and promotes abstinence in PTSD/substance abuse comorbid veterans, and is free of from side effects and withdrawal issues of pharmaceuticals.
- Neurofeedback training demonstrates positive influence in restoring dynamic functional connectivity in the principal resting state network, the Default Mode Network (DNM) as well as improved functional connectivity overall (Lanius et al., 2015). This attests to the positive impact of Neurofeedback on global functioning of the brain. It speaks both to the interconnectivity of neural networks, and neural plasticity of the brain.
- The concomitant release from fear based hyper-vigilance, anchored in the trauma memories embedded in the physiology of the body, can bring positive transformative behavioral and emotional changes.
- The development of the infra low frequency protocols has proven to be a highly successful improvement for the use of Neurofeedback as shown by the success of the last four studies cited above (Othmer and Othmer, 2020).
- Alpha-Theta Neurofeedback training has proven to be an effective method for opening a window into traumatic memories without emotional abreaction. This allows those unresolved memories to be released and processed with less risk of client re-traumatization that is common in talk or exposure therapy (Lake, 2015).
- Neurofeedback has proven to be superior to so-called “evidence-based” PTSD treatment in terms of effectiveness, temporal efficiency, and cost (Fragedakis and Toriello, 2014; van der Kolk, 2014).
- A collateral benefit is the reduced risk of secondary trauma for the clinician.

Collectively, clinical experience has demonstrated that trauma of whatever source can be successfully remediated with Neurofeedback, particularly when ILF NF is combined with more standard EEG-band protocols. Given the success as illustrated above, it is to be hoped that ILF Neurofeedback and Alpha-Theta training become accepted as part of an integrative and holistic approach for treating survivors of trauma.

## Data Availability Statement

The data cited in the concept studies, can be found in the literatures cited. The case study data is shown in Figure 1 and the underlying data are available from the author HK.

## Author Contributions

HK is the principal author. MD is the contributing author. Both authors contributed to the article and approved the submitted version.

## Conflict of Interest

MD was employed by Geers Hypnosis LLC. The remaining author declares that the research was conducted in the absence of any commercial or financial relationships that could be construed as a potential conflict of interest.

## Publisher's Note

All claims expressed in this article are solely those of the authors and do not necessarily represent those of their affiliated organizations, or those of the publisher, the editors and the reviewers. Any product that may be evaluated in this article, or claim that may be made by its manufacturer, is not guaranteed or endorsed by the publisher.

## Footnotes

^1^Reports from multiple persons that in the days after Synchrony sessions they experienced a greater capacity for joy and feelings of being connected to their Higher Self.

^2^Homecoming for Veterans is a world-wide network of clinicians who offer ILF Neurofeedback and Alpha Theta training at no cost, for veterans suffering from PTSD. A directory of participating clinicians can be found at eegfoundation.org.

^3^The Symptom Rating scale developed by the EEG Institute, lists 150 symptom words, divided into seven categories: Sleep, Attention and Learning, Sensory, Behavioral, Emotional, Physical, and Pain. Instructions are to rate each item from 0 to 10, with 10 being very serious or happening most of time. During Intake the client is asked how each of the items scored between 5 and 10 manifests in their life.

^4^A 10-point change in a year is considered significant by the VA.
